# On the evolution of mimicry in avian nestlings

**DOI:** 10.1002/ece3.8842

**Published:** 2022-04-17

**Authors:** Gustavo A. Londoño, Juliana Sandoval‐H, Mohamed F. Sallam, Julie M. Allen

**Affiliations:** ^1^ Facultad de Ciencias Naturales Departamento de Ciencias Biológicas Universidad Icesi Cali Colombia; ^2^ A.C. Red de Biodiversidad y Sistemática Instituto de Ecología Xalapa Mexico; ^3^ Biology Department University of Nevada‐Reno Reno Nevada USA

**Keywords:** Batesian mimicry, Laniisominae, *Laniocera*, masquerade, nest predation, *Schiffornis*

## Abstract

Batesian mimicry (BM), where a nontoxic species resembles a toxic species with aposematic coloring, has been recently described for a Neotropical species of the suboscine passerine (*Laniocera hypopyrra*). Understanding the order and series in which these characteristics evolved is unknown and requires character information from closely related taxa. Here, we trace the origin of mimetic traits and how they evolved by examining antipredator characteristics using images and other field‐collected trait data from nest and nestlings along with data available in the literature for the Laniisominae clade and closely related taxa. We found that morphological modifications of the downy feathers appeared first in the broader clade leading to the Laniisominae clade followed by further morphological and behavioral characteristics within the Laniisominae clade leading to the full BM. Images of nestlings in the Laniisominae and closely related clades demonstrated the extent of antipredator and camouflage characteristics. We found a complex set of behavioral and morphological traits in this clade for reducing predation from hiding to camouflage to mimicry. We further propose the evolution of two distinctive mimicry strategies in the Laniisominae clade: (1) Batesian Mimicry, as described above and (2) Masquerade, resemblance to inedible objects commonly found in their local environment. This complex set of antipredator traits shed light on the diversity of antipredator characteristics in avian nestlings, particularly in neotropical areas where the avian diversity is highest. Unfortunately, there are hundreds of species in the neotropics that lack basic natural history information on nesting traits, and therefore, we are likely missing critical information on the diversity of antipredator characteristics across avian nestlings.

## Graphical abstract

We trace the origin of mimetic traits on a evolutionary novelty, Batesian Mimicry, recently described for a Neotropical species of suboscine passerine (*Laniocera hypopyrra*). We collected anti‐predator trait data from nests and nestlings in the field and combined those with data available in the literature for the Laniisominae clade and sister clades. This information reveals a diverse set of anti‐predatory strategies in nestlings, providing new insights on the nest predation literature in tropical bird communities, which has far‐reaching implications for avian life histories, reproductive behavior, and community composition.

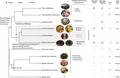

## 1 INTRODUCTION

Understanding how animals’ morphology and behavior change in response to selection from predation remains an important question for evolutionary theory (Ruxton et al., 2018). For birds, nest predation is an important factor affecting avian fitness (Martin, 1993; Ricklefs, 1969; Robinson et al., 2000). Nest predation is not homogeneous for all nestlings and may vary depending on the habitat or on avian characteristics. For example, predation is thought to be higher at lower latitudes and for birds that build open nests (e.g., cup‐shaped; Griebeler et al., 2010; Martin, 1995). In areas with high predation rates, birds exhibit multiple proximal factors that include behavioral, morphological, and physiological traits thought to reduce predation. For example, birds can change their nest location or nest type, as closed nests (e.g., globular or cavity; Griebeler et al., 2010; Martin, 1995) or those located lower in the canopy are thought to be harder to find by predators (Hoset & Husby, 2019; Major et al., 1994; Storaas, 1988). A reduction in the nesting period or number of foraging trips away from the nest limits the exposure time and probability of a predator finding the nest through observing adults flying to the nest (Ghalambor & Martin, 2002; Martin et al., 2007; Robinson et al., 2010; Zanette et al., 2011). Compensatory traits such as increased numbers of nesting attempts or longer nesting seasons may also have evolved in response to predation pressure (Roper et al., 2010).

A highly specialized evolutionary strategy to avoid predation is Batesian mimicry, a phenotypic and sometimes behavioral trait where a nontoxic species resembles a toxic species with aposematic coloring (i.e., true warning coloration; Mallet & Joron, 1999). Batesian mimicry has been documented many times in invertebrates (Guilford, 1990; Iijima et al., 2018; Mallet & Joron, 1999; Ruxton et al., 2018; Schmidt, 1958). However, for vertebrates, only a few cases have been reported (Cheney, 2010; Cox & Davis Rabosky, 2013; Cummings & Crothers, 2013; Dudgeon & White, 2012; Kuchta et al., 2008; Randall, 2005; Ruxton et al., 2018; Twomey et al., 2013), with even fewer found in birds (Caro, 2014; Cott, 1940).

The origin of Batesian mimicry (BM) has long been controversial in evolutionary biology. The debate has included two distinct hypotheses: It has been envisioned either as a cumulative process of trait changes that result in BM (Leimar et al., 2012; Mappes & Alatalo, 1997) or as a multistep process that involves major mutations resulting in BM. The latter could be also seen under the modular evolution hypothesis, where the traits are treated as units that change step by step in a modular fashion (Eble, 2005). This model has been used to explain the morphological and behavioral diversity present in other avian groups (e.g., the enigmatic feather patterns and behaviors of birds of paradise; Scholes, 2008).

**FIGURE 1:**
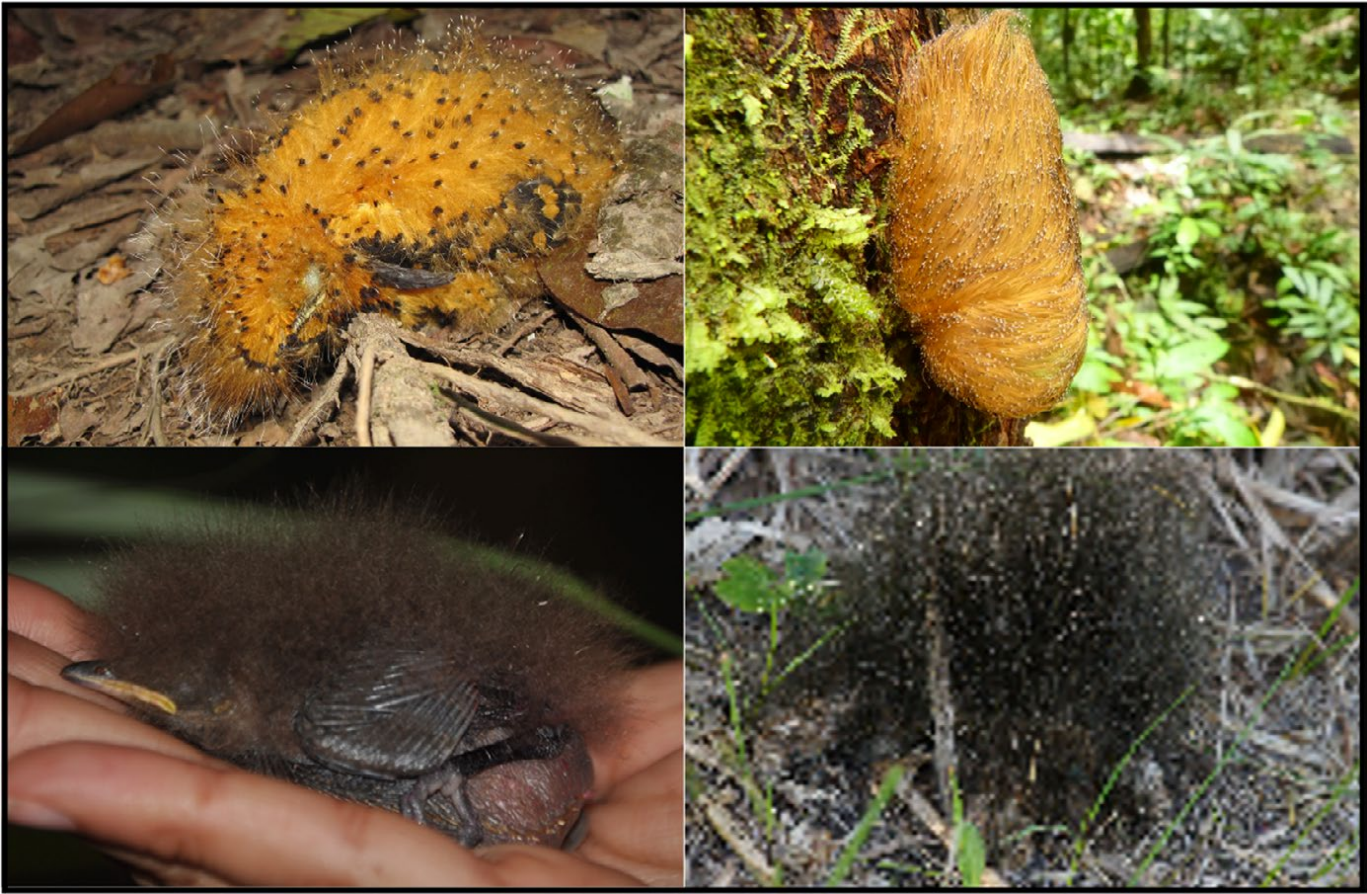
Images of nestlings from the Laniisominae clade. Two forms of mimicry are shown. Top—Batesian Mimicry in nestling of *Laniocera hypopyrra* (left) (Foto by Santiago David). The nestling has many characteristics both morphological and behavioral that increase its similarity to the toxic caterpillar (Megalopygidae) (right) (Foto by Wendy Valencia). Masquerade Bottom—The nestling *Schiffornis turdina* (left) has elongated, dense juvenille downy feathers that resemble a fungus found in the area (right) (Fotos by JSH)

One hypothesized avian case of Batesian mimicry (BM) in nestlings is that of *Laniocera hypopyrra*, a Neotropical bird in the family Tityridae found in subtropical and tropical lowland forests across South America. The nestlings of *L*. *hypopyrra* possess bright orange elongated barbs that mimic caterpillars from the Megalopygidae family (D’Horta et al., 2012; Figure 1), which inhabit the same habitats. These caterpillars are well known for their toxicity (Deml & Epstein, 2001; Dyar & Morton, 1895; Hossler, 2009; Lamdin, 1976; Sánchez et al., 2019), and can be lethal to humans (Pinson & Morgan, 1991). The hypothesis that nestlings of *L*. *hypopyrra* are Batesian mimics is supported by morphological and behavioral traits that enhance the resemblance of nestlings to toxic caterpillars (Londoño et al., 2015). Interestingly, this specific combination of traits in *L*. *hypopyrra* nestlings are not observed in other closely related species (D’Horta et al., 2012; Londoño et al., 2015); in particular, the nestlings do not move or beg in the nest, typical of other nestlings. Furthermore, they move their heads from side to side in a motion that resembles the movements of a caterpillar when they perceive a potential predator (e.g., humans approaching the nest). The morphological traits that enhance their similarity to the Megalopygid caterpillars include increased density, coloration change, and structural modification of the downy nestling feathers (Londoño et al., 2015). The combination of morphological and behavioral traits likely evolved through a complex trajectory of antipredator characteristics resulting in Batesian mimicry (Londoño et al., 2015).

Our goal is to understand in what order these mimetic traits evolved by reconstructing the ancestral character states of *L*. *hypopyrra* and closely related species to shed light on the number of antipredator characteristics found across this group, and to illuminate the way that this mimicry evolved. For example, do we see evidence of the more commonly known antipredator traits for birds (e.g., a decrease in the number of trips to the nest), or did an entirely different evolutionary trajectory occur in these species? How widespread are mimetic traits in this group and in what order do antipredator traits evolve?

Here, we compile morphological and behavioral characteristics of the nestlings from *L*. *hypopyrra* and close relatives to conduct an ancestral character state reconstruction. While some of these morphological features have been known for some time, the nestling behaviors have not been described for many of these species. We combine data from our fieldwork, and from unpublished and published information on morphological and behavioral traits to track evolutionary trajectory of the antipredator traits observed in this clade.

## 2 METHODS

We selected 11 representative species from nine closely related genera based on a recent suboscine passerines phylogenetic reconstruction of this larger clade (Harvey et al., 2020). Species were selected based on the presence of nestling traits in the literature or from our field work. Nestling characteristics are rarely identified and cataloged due to the difficulty in finding nests and collecting these data. In this dataset, we have a single represtative for each genus and this dataset represents the largest set of nestling characterisitics across these clades. From the Laniisominae clade, we included a representative from *Laniocera*, *Laniisoma*, and *Schiffornis*, from its sister clade, Tityrinae; we included the genera *Tityra*, *Pachyramphus* and *Iodopleura*, and from the tribe Onychorhynchini, we included *Onychorhynchus*, *Terenotriccus*, *and Myiobius*, which is the sister clade of the genus *Oxyruncus* (Harvey et al., 2020; Tello et al., 2009; Figure S1). This sampling represents all but a single genus within this larger clade. We used the species *Piprites chloris* a sister species of the family Tityrinae as the outgroup. Most of these species live in sympatry and share the same community of nest predators.

### 2.1 Nesting traits

A total of twelve traits were compiled from published and unpublished data for 11 bird taxa (Table 1; Table S1) representing morphological (modified elongated, dendritic, dense, and juvenile down feathers), behavioral (reduced begging, no movement, and behavioral mimicry), and compensatory traits (length of the nestling period, feeding rate, nest type, nest location, and the number of trips to the nest; Table 1). We also collected pictures of nestlings to examine nestling camouflage found across this clade. We collected missing data from field observations for *Schiffornis stenorhyncha*, *Pachyramphus cinnamomeous*, *Tityra semifasciata*, *Onychorhynchus coronatus*, *Terenotriccus erythrurus*, and *Myiobius atricaudus*. These data were collected, using standardized methodologies found across studies, during a long‐term avian nesting fieldwork project conducted by GAL in Peru and Colombia (see details in the supplementary methods).

**TABLE 1:** Characters collected for downy feathers, behavior, and nest characteristics

Species	Downy Feathers				Behavior			Nesting characteristics				
	Elongated	Dendritic	Dense	Juvenile	Staying still	No begging	Full mimetic	Nestling period	Feeding rate	Nest	Location	Trips
*Tityra semifasciata*	1	1	0	0	0	1	0	24	3.1	Cavity	Both	3.1
*Pachyramphus cinnamomeus*	0	0	0	0	0	0	0	21	8	Globular	Both	8
*Iodopleura pipra*	NA	NA	1	0	1	1	0	NA	4.1	Cup	Canopy	NA
*Laniocera hypopyrra*	1	1	1	1	1	1	1	20	1	Cup	Understory	1
*Laniisoma elegans*	1	1	1	1	NA	NA	NA	NA	NA	NA	NA	NA
*Schiffornis turdina*	1	1	1	1	1	1	0	20	1	Cup	Understory	1
*Oxyruncus cristatus*	**0**	0	1	0	**0**	**0**	0	26	1.5	Cup	Canopy	1.5
*Terenotriccus erythrurus*	0	0	0	0	0	0	0	23	NA	Globular	Understory	3.3
*Myiobius atricaudus*	0	0	0	0	0	0	0	21	NA	Globular	Understory	7
*Onychorhynchus coronatus*	0	0	0	0	0	0	0	20	NA	Globular	Canopy	5
*Piprites chloris*	NA	NA	NA	NA	NA	NA	NA	NA	NA	Cavity	Understory	NA

Note

Downy feather charactersitics include the presence of elongated, dendritic, dense, and juvienile feathers. Behavior characteristics indicate if the nestlings stay still when the nest is approached and do not beg and if they are full Batesian Mimics—where they include all of the characteristics. Nestling characteristics include Nest type (cup, cavity, or globular), location of the nest. Nestling period is in Days (we took the median if more than one period was available), Feeding Rate is (Trips/hour to the nest with food) and Trips (Trips/hour to the nest).

### 2.2 Ancestral character state reconstruction

To reconstruct the ancestral character states, we obtained a phylogenetic tree from Harvey et al. (2020), which included all the species in this study, and pruned it to our 11 taxa (see Figures S1–S4). This topology does not differ from other studies focused on species within these groups (Prum & Lanyon, 1989; Tello et al., 2009). We further downloaded 1000 trees from birdtree.org (Jetz et al., 2012) to examine uncertainty across the distribution of trees. The major clades were identical across the 1000 trees apart from the placement of *Oxyruncus*. These taxa were placed in different clades but never within the Laniisominae or Tityrinae clade. Furthermore, running the analysis on all 1,000 trees did not change the results of the major nodes represented in the results (data not shown). We note that the species represented in our tree as *Schiffornis stenorhyncha* has been split into five species (Chesser et al., 2013; Nyári, 2007; Remsen et al., 2022). However, these species are not included as independent branches in the tree, rather they are presented as a single branch (*Schiffornis stenorhyncha*), the overall tree topology with relationship to the other genera is not changed and the nesting traits known for the five species do not vary.

To determine the trend and order of the evolution of these traits, we first determined the best fit model of evolution for each character and, secondly, ran an ancestral character state reconstruction to determine the character state at each ancestral node. With the character state identified at each node, we then determined the progression of character states leading to Batesian Mimicry in this group. We used Geiger (Pennell et al., 2014) and Phytools (Revell, 2012) libraries in R statistical packages to model the evolution of characters and reconstruct the ancestral character states. The phylogenetic tree was visualized and generated using ggtree (Yu et al., 2017) and plotrix (Lemon, 2006) packages in R statistical software (R Core Team, 2021).

#### 2.2.1 Discrete character states

The nine discrete data included both binary (1,0; present, absent) and multistate (1,2,3) characters (Table 1). We evaluated three models to determine which was the best fit for each character. The models included (i) Equal Rates “ER”: the rate of transitions between character states are equal (i.e., rate of evolution from state 0 to state 1, and vice versa), (ii) All‐Rates‐Different “ARD”: in which the rates of transitions between character states backward & forward are different, and (iii) Symmetric Entities “SYM”: where the forward and reverse transitions between character states are symmetric (i.e., the probability of changing from 0 to 1 is the same as 1 to 0) (Cadotte & Davies, 2016). We then compared the overall corrected Akaike Information Criterion (AICc) values from all three models for each character to select the best model. For all characters, we found the best fitting model was ER and used this model in the ancestral character state reconstructions.

The Ancestral Character Reconstruction (ACR) model assigns a character state for each ancestral node that gives the highest correlation given the character states and the rate of change in the character. We examined the character state assignments and probabilities for each node and assigned the character state for the probability over 0.5 for binary states and 0.33 for the multistate characters (all included only three states).

#### 2.2.2 Continuous character states

We first determined which model of evolution had the best fit for the three continuous characters using four different models: (i) Early Burst (EB): high early rates of change that slow exponentially through time, (ii) Brownian Motion (BM): the expected amount of character change along a branch is zero, (iii) BM with white noise: similar to BM except that ignores the phylogenetic structure, and (iv) BM with a trend (i.e., drift) in the mean trait value: the trait value increases or decreases indefinitely.

We used the Fast Ancestral (FastAnc) model to estimate the characters for the nodes and the confidence intervals at 95%. Fast estimation of the Maximum likelihood (ML) examines the character states at the internal nodes in the tree and finds the parameter values that maximize the probability of the data on the tree. We examined the estimated ancestral states to determine whether they were higher or lower than the current states to understand how these characters have changed overtime.

## 3 RESULTS

We were able to collect data for most traits for all of the taxa either from the literature or from the field (Table 1). *Iodopleura isabellae* presented the largest challenge with the fewest characters in the literature. This species nests in the canopy, which makes finding and monitoring nests particularly difficult and likely explains the lack of nesting data.

### 3.1 Down feather modification

**FIGURE 2:**
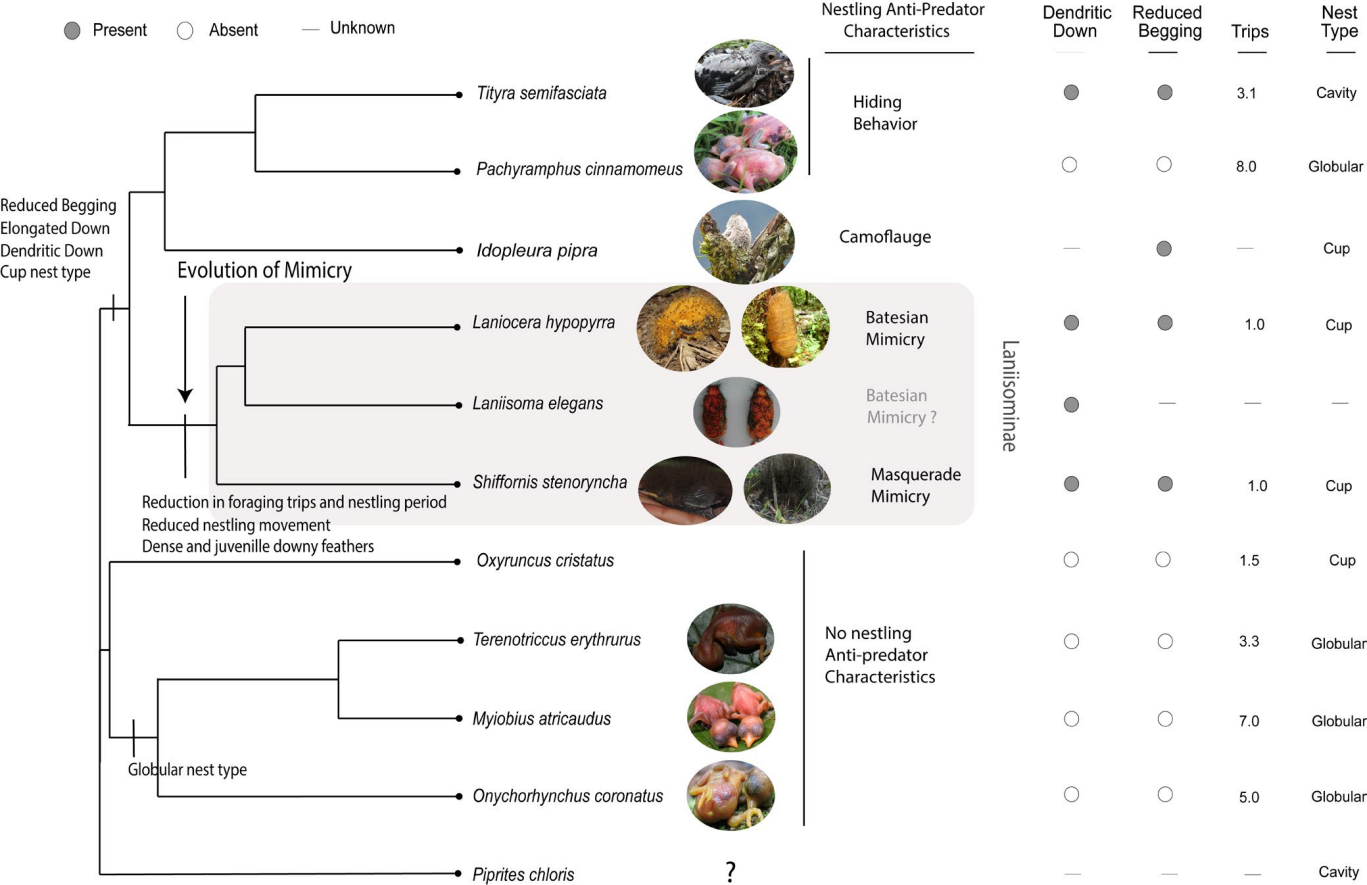
Phylogenetic tree showing the possible origin of each trait according to the ancestral character reconstruction. It also presents the presence, absence, or value of each Batesian mimicry related trait for each species. All the photographs were taken during GAL diferent projects except the nestlings of Idopleura pipra taken by Francisco Piedrahita, Laniisoma elegans modify from D’Horta et al. (2012) Figure 2 and the nestling of Oxyruncus cristatus taken by Walter Coto

*Schiffornis turdina* (Skutch & Eckelberry, 1969) and *S*. *stenorhyncha* nestlings were completely covered with a dense brownish down, with a few elongated dendritic barbs of the same color (Figure 2), sharing with *L*. *hypopyrra* the density and elongation of the down barbs. For *T*. *semifasciata*, we obtained information from Skutch and Eckelberry’s (1969) observations that describe the presence of dendritic down for nestlings with “fairly long light gray down” on head, wings, and back that did not entirely cover the pink skin. By contrast, *Pachyramphus polychopterus* (Skutch & Eckelberry, 1969) and *P*. *cinnamomeous* (this study) nestling down is lacking. For *S*. *stenorhyncha*, the dendritic downy feathers remained until the nestlings fledged notwithstanding having feathers all over the body, this can also be seen in *Laniisoma* and *Laniocera* juveniles (D’Horta et al., 2012) but not in *T*. *semifasciata* and *P*. *cinnamomeous* (Skutch & Eckelberry, 1969; Figure 2). Furthermore, down length and complexity are shared by the Laniisominae clade with at least one species of its sister clade. The high density of downy feathers and their retention after fledging are only present within the Laniisominae clade. Most of the nestlings of species in the Tityiridae have no down feathers upon hatching (Figure 2).

#### 3.1.1 Nestling behaviour

We know that the behavioral mimicry in *L*. *hypopyrra* is exhibited only during a portion of the nestling period (Londoño et al., 2015). During most visits to the nest, *S*. *stenorhyncha* nestlings stood still exhibiting no movement and did not beg for food, which was accompanied by lowering the head toward the belly when the researchers approached the nest. Similarly, Skutch and Eckelberry (1969) describe that when they approached and shook the nest of *S*. *turdina* or held a hand above the nestlings, they did not lift or gape their mouth as many young nestlings do, they remain motionless. These behaviors were not observed during the whole nestling period of *S*. *stenorhyncha*. Therefore, it is possible that some of these behavioral characteristics are only present during a portion of the nestling period similar to *L*. *hypopyrra* (Londoño et al., 2015). Skutch and Eckelberry (1969) also described that the no begging and no movement behavior of *T*. *semifasciata* nestlings changed overtime. In *T*. *semifasciata* and *P*. *cinnamomeous*, a different suite of behavioral traits may reduce predation: These birds hide inside their closed nest when researchers approached. Begging and movement behavior was always present when checking the nests of these species.

*Nest Type and Adult Behaviour*: The feeding rates observed in the Laniosoma clades, one trip per hour in *S*. *stenorhyncha*, *S*. *turdina*, and *L*. *hypopyrra* are low compared with other species from the family and sister clades according to Skutch and Eckelberry’s (1969) (See also Figure 2). These species also have open cup nests, which are thought to be particularly vulnerable to predation. Related clades have closed nests and much higher feeding rates that are within the range found in other Neotropical Passeriformes (Martin et al., 2011) that average 5 feedings per hour. Yet, the nestling period in the Laniisominae clade is within the range of the other clades.

### 3.2 Traits within other members of the Laniisominae clade

For all of the nine discrete traits, the Equal Rates (ER) model was found to be the best fit, and for the three continuous traits, the Brownian Motion (BM) model was found to be the best fit for each character. Through ACR of traits, we were able to assess that the presence of elongated down, dendritic down, reduced nestling begging, and the cup‐shaped nest appeared in the ancestor of the clade that contains the genera *Laniisoma*, *Laniocera*, *Schiffornis*, *Iodopleura*, *Pachyramphus*, and *Tityra* (Figure 2). Further mimetic traits were evolved along with the ancestor to the Laniisominae clade including dense and juvenile downy feathers, further reduced nestling movement, and a reduction in the frequency of food provisioning, suggesting high predation pressure along this lineage. Finally, bright downy coloration is present in the clade formed by *Laniisoma* spp. and *Laniocera* spp., but the lack of information concerning *Laniisoma* does not allow us to assert whether the caterpillar‐like movement is present in both species and if this species is a full Batesian mimic (Figure 2). Interestingly, *Tityra* spp. and *Pachyramphus cinnamomeous* nestling use a different strategy to reduce predation, they hide under the dry leaves in their cavity (*Tityra* spp.) or the back of their globular nest (*Pachyramphus* spp.). Globular and cavity nests are dark; hence, down coloration is not important, but hiding behaviors could play an important role.

## 4 DISCUSSION

Our results suggest that the unusual phenotypic traits involved in Batesian mimicry in the Neotropical forest bird *Laniocera hypopyrrha* and its relatives evolved in a gradual stepwise fashion made possible by a series of steps starting with behavior and down feather modifications and later further behavior and plumage modifications. The nestlings of *S*. *stenorhyncha* are a possible undescribed case of mimicry among neotropical nestlings. The downy feathers are elongated and dense but are black in color. These nestlings also have reduced nestling movement as well. Here, we propose a different kind of antipredator strategies for this lineage, Masquerade (Caro, 2014; Skelhorn et al., 2010), where the mimetic organism resembles objects of no particular interest to the predator, such as rotten fruit (Snow, 1982). In this case, the nestlings of *Schiffornis stenorhyncha* resembles a fungus found in the area (Figure 1). This suggests that mimicry may have evolved in the ancestor of the Laniisominae clade. We suggest that a key step toward the Batesian mimicry was the origin of downy nestling plumage, which opened the door for two different kinds of antipredator mimicry strategies in this clade, Masquerade, and Batesian Mimicry (Figure 2).

The risk of predation is higher in cup nesters than for those who nest in cavities or globular nests observed in the sister clades (Arendt, 1997; Martin & Li, 1992; Oniki, 1979; Ricklefs, 1968; Wilcove, 1985). *L*. *hypopyrra* and *S*. *stenorhyncha* are cup nesters, which likely increased predation pressure on those nests and may be the reason further antipredator traits were selected for in *Laniocera* and *Shiffronis* including the reduction in the number of visits to the nests and feeding rate (Martin et al., 2000). The slow feeding rate could also be affecting the growth rate (Crossner, 1977; Ricklefs, 1968), increasing the nestling period (Richner et al., 1989). Martin et al. (2011) found, when predation pressure was strong, an increase in growth rates with lower food delivery in passerine birds, at the expense of reducing phenotypic attributes (e.g., smaller wings). Our results suggest an alternative explanation to Martin et al. (2011) hypothesis; we propose that parental investment and behaviors are not the only mechanisms to cope with high predation in the tropics, nestling behavior and morphology can play an important role in reducing predation risk (Trnka et al., 2016). The specialized nestling phenotype likely resulted from the combination of a long nestling period and high predation risk in the Laniisominae clade. Across the Laniisominae clade, the nestlings have reduced begging, reduced feeding trips to the nestlings, and further modification of the dense and juvenile downy feathers; therefore, adaptive coloration and antipredator strategies may be an important strategy across this clade to reduce predation in an open cup‐shaped nest.

Other mimetic traits have been observed in species from this clade, including nestling camouflage (Snow, 1982) and hiding among dead leaves or in the back of nests (rather than typical begging behaviour), suggesting antipredator behaviours, reduce begging, elongated down, dendric down, have been common and evolved early in this clade (Figure 2) corresponding to our finding of these characteristics at the base of this clade. Some studies suggest that certain tropical birds in lowland and humid forests do not have longer breeding seasons or more nesting attempts per year (Gill & Haggerty, 2012); therefore, species in this clade may not rely on some of the more commonly found antipredator characteristics and may have evolved different nesting traits that could increase their reproductive success in areas with high nest predation such as the lowland and humid forests.

The main constraint to tracking the evolution of these traits in *Laniocera* is the lack of natural history information from closely related species. In our sample, we have a single representative across these genera making this a relatively small sample size. This limitation is likely the reason we found that Brownian Motion was the best fit model for the contiuous data. Brownian Motion suggests neutral evolution; however, we argue that these extreme rare traits (e.g., bright orange coloring and non‐begging nestlings) are more likely the result of selection and our result is due to the small sample size. For example, we know that the sister taxon to *L*. *hypopyrra*, *Laniisoma elegans*, has some of the morphological traits exhibited by *L*. *hypopyrra*, including the bright orange coloration and structural downy feather modifications, but whether nestlings display behavioral mimicry is unknown (see details on Londoño et al., 2015). Some information on the downy characteristics and coloration is known for the sister genus *Schiffornis* (Sandoval‐H et al., 2017; Skutch & Eckelberry, 1969). For example, the downy feather of *Schiffornis veraepacis* and *S*. *stenorhyncha* are brownish‐gray and more abundant than that of nestlings of the majority of passerine birds (Sandoval‐H et al., 2017; Skutch & Eckelberry, 1969), and the latter has dense down composed of elongated dendritic barbs, suggesting that peculiar feather structures are present in *Schiffornis*, and may play an important role in its behavioral mimicry. Why might downy nest plumage have evolved? Could it be a defense trait on its own, or could it have evolved for some other function, which in turn enabled new defense adaptations (e.g., color) to be effective? These are unresolve questions that need further exploration.

Our findings unraveled an extraordinary diversification of antipredatory strategies in the Laniisominae–Tityrinae clade. For example, most nestlings in this clade, with known behaviour, have reduced begging behavior relative to other birds. *T*. *semifasciata* nestlings hide under dry leaves on their cavity nest, *P*. *cinnamomeus* nestlings hide in the back of their globular nest. Unfortunately, there is no information on recently hatched nestlings for *Idopleura* spp., but, as described by Willis and Oniki (1988), the old nestling of *I*. *pipra* has the color and the irregularity of the dorsal plumage, resemble a scaly mass of bark or a bulky mass of debris on the flattened nest, so that it was not obvious when it sat quietly, as it did much of the day. This behavior and coloration of the old nestling of *I*. *pipra* match the coloration and feather pattern of old nestlings from the genus *Nyctibius* that uses cryptic traits to reduce predation. Additionally, *Idopleura* spp. has a very small nest for their size and presumably one egg clutch size (Ingels & Vinot, 2010; Snow, 1982; Whittaker & Kirwan, 2008; Willis & Oniki, 1988), traits associated with high predation.

Furthermore, the behavioral trait evolved in a similar way where Laniisominae sister clade nestling exhibit a hiding behavior inside the nest, while the nestlings of *S*. *stenorhyncha* and *S*. *turdina* (Skutch & Eckelberry, 1969) exhibit a no movement behavior and the *L*. *hypopyrra* nestlings exhibit a complex head movement when in danger. Taken together, these results suggest a complex set of behaviors and traits in this clade limiting predation from hiding and camouflage to Masquerade and Batesian mimicry, expanding our ideas of the possibilities and lability of antipredator behaviors in avian nestlings, mainly in neotropical areas where the avian diversity is the highest. It is likely that the evolution of mimetic and antipredatory strategies observed in this group occurred under the selection pressure of a community of predatory species, rather than a single predator species that impose a high predation pressure (Schmidt, 1958). Yet, this may only be important for species that build open nests (e.g., cup and platforms), as few nest predators have access to close nests (e.g., cavity and globular).

Unfortunately, there are hundreds of bird species in the neotropics that lack basic natural history information on nesting traits (Xiao et al., 2017). Hence, we encourage naturalists to explore the neotropical forest in search of the unknown nests that could uncover amazing traits such as the adaptive coloration and diverse antipredatory strategies observed in this group.

## CONFLICT OF INTEREST

We do not have any conflict of interest.

## AUTHOR CONTRIBUTIONS

**Gustavo A. Londoño:** Conceptualization (equal); Data curation (equal); Funding acquisition (lead); Investigation (lead); Methodology (equal); Project administration (lead); Resources (lead); Supervision (equal); Writing – original draft (equal); Writing – review & editing (equal). **Juliana Sandoval‐H:** Conceptualization (equal); Data curation (equal); Formal analysis (supporting); Investigation (supporting); Visualization (supporting); Writing – original draft (equal); Writing – review & editing (equal). **Mohamed F Sallam:** Data curation (equal); Formal analysis (equal); Methodology (equal); Software (equal); Writing – review & editing (equal). **Julie M. Allen:** Conceptualization (supporting); Data curation (lead); Formal analysis (lead); Methodology (equal); Software (lead); Visualization (equal); Writing – original draft (equal); Writing – review & editing (equal).

## Supporting information

Appendix S1

## Data Availability

All the raw data are avalible on the Appendix S1 tables.
