# Supplementary material for: On the evolution of mimicry in avian nestlings
Source: Ecol Evol. 2022 Apr 17;12(4):e8842. doi: 10.1002/ece3.8842 (PMC9013854; doi:10.1002/ece3.8842)
Supplement: Supplementary file 1 — Appendix S1 [file ECE3-12-e8842-s001.docx]

APPENDIX

**Supplementary Methods**

**Nesting traits**

The Peru data were collected between 2008 and 2014 from August through December on an elevational gradient between 300 and 3100 m. Specifically, the nesting data for *Onychorhynchus coronatus*, *Terenotriccus erythrurus* and *Myiobius atricaudus* were collected at the Pantiacolla Lodge (12.6420288 S, 71.2392228 W; 412 m elevation) and Tono River (12.9562228 S, 71.4816398 W; 950 – 1,000 m elevation), see Londoño et al. (2017) and Sánchez-Martínez et al. (2017) for further details. The fieldwork in Colombia occurred between February to June. The *Pachyramphus cinnamomeous* nest was found and monitored in 2019 at the Anchicayá station that is located in the Choco region (3.5621667 N, -76.8765 W; 580 m) and the *Schiffornis stenorhyncha* nest was found and monitored in 2014 at a site located in Remedios, Antioquia (6.96675 N, -74.765425 W; 550 m; see details on Sandoval-H et al. 2017). Once a nest was found, we identified the species through direct observations or video recordings. The mass of the nest, eggs, and nestling were taken using a pocket scale (0.01 g accuracy; TRITON T2, Phoenix, AZ, USA) and morphological measurements were taken using a caliper (Swiss Precision Instruments, Garden Grove, CA, USA, INC-2000, 0.01 mm accuracy). Nestlings were weighed and measured every other day and the downy feathers examined for these modifications. The activity of the nestlings was monitored and recorded as researchers approached the nest. The activity of the parents at the nest during the incubation stage was monitored with a U-12 HOBO data logger (Onset Computer Corporation, Pocasset, MA, USA, http://www.onsetcomp.com), recording inner nest temperature every minute and/or a motion camera trap (Reconyx PC85 or R60 Rapidfire professional colour IR) that took ten photos when movement occurred at the nest and one photo every minute when no movement was detected. The parental behaviours at the nestling stage were monitored with a camera trap.

Table 1S. Sources of each especific trait analized in this study.

Supplemental Figures


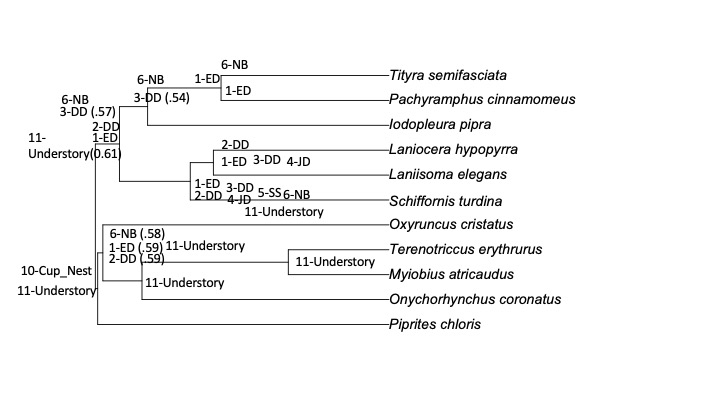


Supplemental Figure 1. All discrete character ancestral states are mapped onto the nodes. The probability is only indicated in cases where it was less than 0.70. For each character where the character was indicated as present is mapped. Characters and character states: 1 – Elongated down, 2-Dense Down, 3 – Dendritic Down, 4 – Juvenile Down, 5 – SS – no movement in the nest, 6 – NB - Non-Begging in the nest, 10 – Nest type (cup, globular, Cavity), 11 – Nest location (Understory, Canopy, Both).

Supplemental Figures 2-4. Continuous character state mapping. The character states are written at the tips and the ancestral states indicated next to the node number. Figure 2: character 8 - Nestling Period (Days). Figure 3: Character 9 – Reduced Feeding Rate (Trips/hour). Figure 4: Number of off-bout trips from the nest during incubation (Trips/hour).

**Appendix references**

Camacho, A, E. Biamonte, L. Sandoval, and C. Sánchez. 2010. Sharpbill *Oxyruncus cristatus* frater nesting ecology in Costa Rica. Cotinga 32: 69–71.

D’Horta, F. M., G. M. Kirwan, and D. Buzzetti. 2012. Gaudy juvenile plumages of Cinereous Mourner (*Laniocera hypopyrra*) and Brazilian Laniisoma (*Laniisoma elegans*). The Wilson Journal of Ornithology 124:429–435.

Ingels, J., and A. Vinot. 2010. First nest of Dusky Purpletuft *Iodopleura fusca*, from French Guiana. Bulletin of the British Ornithologists’ Club 130:71–72.

Londoño, G. A., D. A. García, and M. A. Sánchez Martínez. 2015. Morphological and behavioural evidence of Batesian mimicry in nestlings of a lowland Amazonian bird. The American naturalist 185:135–141.

Londoño, G. A., M. A. Chappell, J. E. Jankowski, and S. K. Robinson. 2017. Do thermoregulatory costs limit altitude distributions of Andean forest birds? Functional Ecology 31:204–215.

Sánchez-Martínez, M. A., S. David, G. A. Londoño, and S. K. Robinson. 2017. Brood parasitism by the enigmatic and rare Pavonine Cuckoo in Amazonian Peru. The Auk 134:330–339.

Sandoval-H, J., G. A. Chinome, and G. A. Londoño. 2017. Nesting biology of *Schiffornis stenorhyncha* (Tityridae). The Wilson Journal of Ornithology 129:827–833.

Skutch, A. F. 1960 Life histories of Central American birds II: Families Vireonidae, Sylviidae, Turdidae, Troglodytidae, Pariidae, Corvidae, Hirundinidae, and Tyrannidae. Cooper ornithological society.

Skutch, A.F., and D. R. Eckelberry. 1969. Life Histories of Central American Birds: Families Cotingidae, Pipridae, Formicariidae, Furnariidae, Dendrocolaptidae, and Picidae. III. Cooper Ornithological Society.

Whittaker, A. and Kirwan, G. M. 2008. Natural history data for the canopy-dwelling purpletufts *Iodopleura* (Cotingidae), and first documentation of Dusky Purpletuft *I. fusca* for Brazil. Bulletin of the British Ornithologists’ Club 128: 28–35.
